# Noninvasive Pressure-Volume Loops: Can Cardiac MRI Obviate the Need for Invasive Catheter Hemodynamic Measurements?∗

**DOI:** 10.1016/j.jacadv.2024.101000

**Published:** 2024-05-16

**Authors:** Kate Hanneman, Gaurav S. Gulsin

**Affiliations:** a Department of Medical Imaging, University of Toronto, Toronto, Ontario, Canada; b Joint Department of Medical Imaging, University Health Network (UHN), University Medical Imaging Toronto, Toronto, Ontario, Canada; c Toronto General Hospital Research Institute, University Health Network (UHN), University of Toronto, Toronto, Ontario, Canada; d Department of Cardiovascular Sciences, University of Leicester and the NIHR Leicester Biomedical Research Centre, Glenfield Hospital, Leicester, United Kingdom

**Keywords:** cardiac MRI, heart failure, heart failure with reduced ejection fraction, pressure-volume loops

**Figure:**
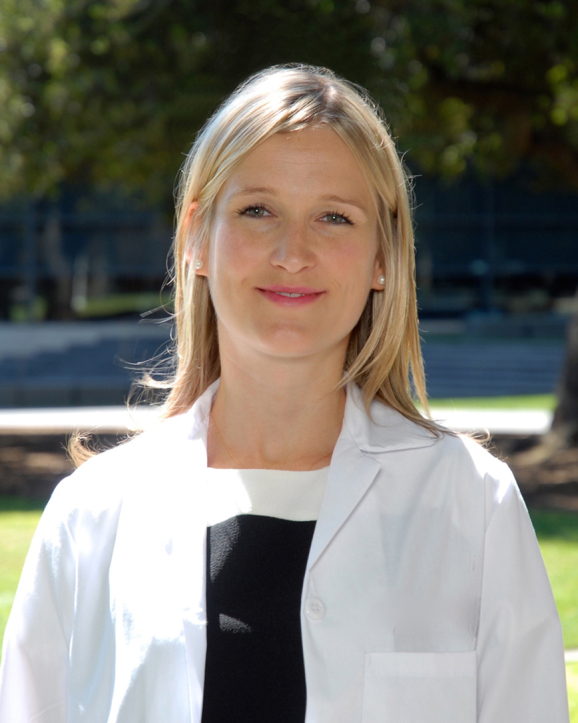

Pressure-volume (PV) loops visually display the relationship between left ventricular pressure and volume across the cardiac cycle. Aspects of ventricular function and cardiac mechanics that are not readily available by other means, such as stroke work and contractility, can be quantified using PV loop analysis.^1^ These metrics are clinically relevant in the growing population of patients with heart failure, both as a tool to improve characterization of cardiac function in clinical practice and as a potential surrogate end point in clinical trials.^2^

However, a major barrier to routine clinical implementation is that PV loop measurements have historically required invasive assessment of left ventricular pressures using catheterization. Such measurement involves placement of a PV conductance transducer with tip in the left ventricular apex and proximal end in the aortic root. This allows for simultaneous recording of pressure and volume. However, these invasive measurements are costly and time-consuming, require specific technical expertise, and have inherent associated procedural risks.

Therefore, noninvasive imaging surrogates for invasive physiology and alternate methods for PV loop estimation are highly desirable. Prior studies that explored imaging estimates of ventricular PV relationships utilizing resting transthoracic 2-dimensional echocardiography demonstrated modest correlations between echocardiographic parameters and invasive filling pressures.^3^ More recently, noninvasive PV loop estimates have been described using an elastance model with inputs of left ventricular volume curves and heart rate from cardiac magnetic resonance imaging (MRI) along with left ventricular peak pressure estimated from brachial pressure.^2^ This approach has been previously validated in porcine models using invasive measurements and applied in small cohorts of human patients.^2^

In this issue of *JACC: Advances*, Arvidsson et al^4^ advance our understanding of the potential utility of cardiac MRI-derived PV loops with respect to prediction of major adverse cardiac events (MACE) in patients with heart failure with reduced ejection fraction (HFrEF). This retrospective cohort study evaluated 164 patients with a clinical diagnosis of heart failure, cardiac MRI with brachial blood pressure measurement between 2004 and 2014, and left ventricular ejection fraction ≤40%. Median age was 63 years [IQR: 55-70 years], 79% were male, 60% had New York Heart Association functional class II or III symptoms, median N-terminal pro b-type natriuretic peptide was 1,567 μg/L [IQR: 596-3,160 μg/L], mean left ventricular ejection fraction was 26% ± 8%, and late gadolinium enhancement was present in 78%. Underlying etiology of HFrEF was classified as ischemic cardiomyopathy in 55% and nonischemic dilated cardiomyopathy in 45%.

PV loops were evaluated using a digitized time-variance elastance function to estimate dynamic left ventricular pressures across the cardiac cycle in relation to left ventricular volumes derived from analysis of cardiac MRI cine steady state free precession images (Figure 1). Briefly, the elastance model is scaled both in time (so that left ventricular end-systolic volume coincides with the midpoint of the elastance function downslope) and in amplitude (to match left ventricular peak pressure and end-diastolic pressure). Of note, the left ventricular end-diastolic pressure input is estimated by the user and, in this study, was set to 7 mm Hg for all. Multiple measures of cardiac mechanics were derived from the resulting PV loops, summarized in Figure 1.

**Figure 1:**
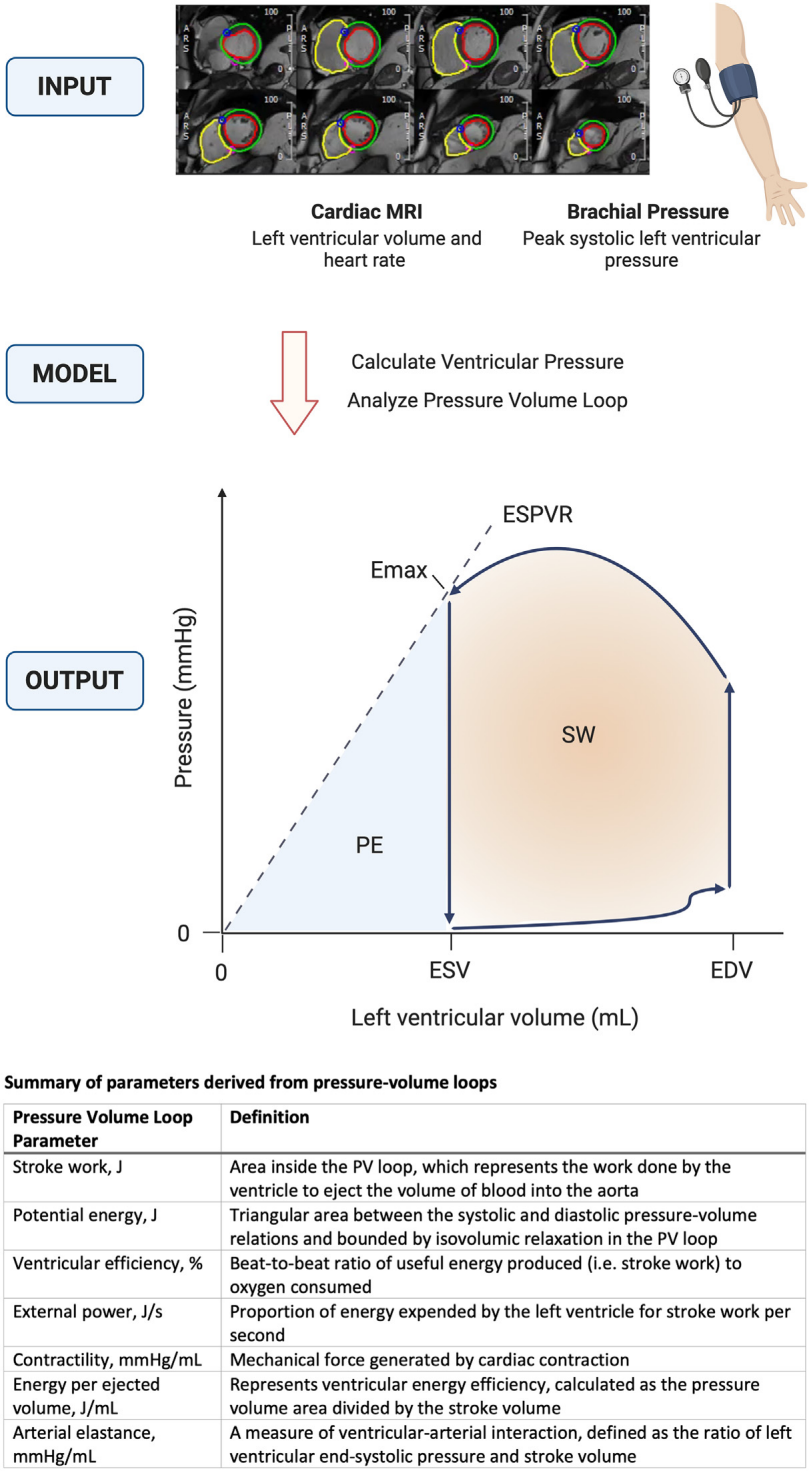
**Cardiac MRI-Derived Pressure-Volume Loops** Input includes left ventricular volumes and heart rate derived from cardiac MRI cine balanced steady state free precession images and peak left ventricular systolic pressure from brachial blood pressure. The elastance is modelled from these data with calculation of left ventricular pressure. Output includes analysis of the pressure-volume loop.^2^ The light orange area enclosed by the loop is the stroke work (SW). The light blue area bounded by the PV loop and the end-systolic pressure volume relationship (ESPVR) is potential energy (PE). Ventricular efficiency (VE) is calculated as: SW/(PE + SW). ESPVR is defined as the slope from the origin to the point of maximal elastance (Emax). Created with BioRender.com. EDV = end-diastolic volume; ESV = end-systolic volume; MRI = magnetic resonance imaging; PV = pressure-volume.

Clinical follow-up was via evaluation of electronic hospital records and national death registry data, for an average duration of 4.5 years. The primary outcome was incident MACE, defined as a composite of cardiovascular death, heart failure hospitalization, myocardial infarction, coronary revascularization, cardiac arrest, pulmonary edema, heart transplantation, sustained ventricular tachycardia or ventricular fibrillation, or left ventricular assist device treatment. MACE occurred in 88 individuals (54%) at average 2.8 years. Associations between baseline cardiac MRI-derived PV loop parameters and MACE were evaluated using univariable and multivariable time-to-event survival analysis with Cox proportional hazard models.

In univariable models, several of the PV loop parameters were associated with MACE including stroke work, ventricular efficiency, external power, contractility, and energy per ejected volume. In a multivariable model adjusted for age, sex, hypertension, and diabetes, the only PV loop parameter that remained a significant predictor of MACE was ventricular efficiency (HR: 1.04 per 1% decrease in ventricular efficiency; 95% CI: 1.01-1.08, *P* = 0.01). Ventricular efficiency reflects the mechanical energy generated by left ventricular contraction, a function of stroke work and mechanical potential energy.

Strengths of the current analysis include prognostic data in a relatively large cohort of patients with HFrEF and long-term follow-up. Limitations include the retrospective study design, heterogenous patient population, lack of invasive measurements for validation, rest only cardiac MRI without preload manipulation, lack of reproducibility analysis, and a broadly defined composite endpoint. Of note, left ventricular ejection fraction and ventricular efficiency are geometrically related and highly correlated. To avoid multicollinearity, the prognostic value of left ventricular ejection fraction and ventricular efficiency were not compared and therefore the incremental value of cardiac MRI-derived PV loop metrics is unknown.

The authors should be commended for advancing our understanding of the potential prognostic utility of noninvasively derived PV parameters. Herein lies the major potential of this technique for PV loop estimation; that it may be expanded to cohorts beyond HFrEF by leveraging previously acquired cardiac MRI images for opportunistic evaluation of cardiac functional parameters that are not routinely reported. A prior study estimated left ventricular filling pressure from cardiac MRI in patients with suspected heart failure, supporting the potential clinical utility of such approaches.^5^ Notably, the analysis to derive PV loop metrics from cardiac MRI images is time-consuming and requires dedicated software and expertise which is an impediment to clinical implementation. However, artificial intelligence applications with in-line automated analysis of PV loop parameters could conceivably be integrated into existing cardiac MRI post-processing software workflows.^6^ Future studies should evaluate the incremental value of PV loop parameters to ejection fraction and expand beyond patients with HFrEF to include heart failure with preserved ejection fraction, valvular heart disease, and pulmonary hypertension. Leveraging information from routine noninvasive imaging has the potential to obviate the need for invasive assessments, reducing cost, risk, and environmental impact.^7^^,^^8^

## Funding support and author disclosures

The authors have reported that they have no relationships relevant to the contents of this paper to disclose.
